# Effects of Continuous Low-Level UV-B, Alone or in Combination with Blue Light, on Photosynthetic and Antioxidant Responses of Morphologically Distinct Red-Leaf Lettuce Cultivars

**DOI:** 10.3390/plants14243821

**Published:** 2025-12-16

**Authors:** Ivan A. Timofeenko, Mikhail Vereshchagin, Ekaterina Dranichnikova, Nikolay Sleptsov, Anna Abramova, Olga V. Buyko, Arina Manevich, Vladimir Kreslavski, Pavel Pashkovskiy

**Affiliations:** 1 Interdisciplinary Laboratory of City Farming, Institute of Gastronomy, Siberian Federal University, 660041 Krasnoyarsk, Russia; katya.gromova96@mail.ru (E.D.); obuyko@sfu-kras.ru (O.V.B.); manevicharina@gmail.com (A.M.); 2 K.A. Timiryazev Institute of Plant Physiology, Russian Academy of Sciences, 127276 Moscow, Russia; mhlvrh@mail.ru (M.V.); ann.kiedis2000@gmail.com (A.A.); pashkovskiy.pavel@gmail.com (P.P.); 3 Department of Plant Physiology, Russian State Agrarian University, 127550 Moscow, Russia; inkss@mail.ru; 4 Institute of Basic Biological Problems, Russian Academy of Sciences, 142290 Pushchino, Russia; vkreslav@rambler.ru

**Keywords:** *Lactuca sativa* L., continuous low-intensity UV-B, photosynthetic performance, PSII quantum yield, antioxidant capacity, phenolic metabolism, photoprotection, light signaling, cultivar-specific adaptation, controlled environment agriculture

## Abstract

The physiological, biochemical, and morphometric responses of two lettuce cultivars (*Lactuca sativa* L.), Gypsy and Pomegranate Lace, which differ in terms of leaf morphology and anthocyanin pigmentation, were examined under moderate light (290 µmol m^−2^ s^−1^) with the addition of blue light (BL, peak at 450 nm), UV-B (peak at 306 nm), and their combinations. Continuous low-intensity UV-B (30 mW m^−2^) was applied for 48 h—during the day with white (WL, Red: 51%, Green: 38%, Blue: 11%) or white + blue (WL + BL, Red: 30%, Green: 22%, Blue: 48%) light and at night alone—to assess the effects of sustained UVR8 activation in the absence of visible light. In the Pomegranate Lace cultivar, which has wrinkled leaves and localized anthocyanin pigmentation, the combination of WL + BL + UV-B enhanced the chlorophyll and carotenoid contents, photosynthetic rate, and stomatal conductance, whereas respiration did not change. These coordinated changes indicate efficient integration of cryptochrome and UVR8 signaling, which sustains photochemical efficiency and stimulates phenolic and carotenoid accumulation, reinforcing antioxidant capacity. In the Gypsy cultivar, which is characterized by smooth leaves and uniform pigmentation, UV-B + BL increased g_S_ along with the rates of respiration and photosynthesis and improved PSII efficiency. However, both cultivars showed a decrease in biomass and leaf area. Nevertheless, both cultivars exhibited increased antioxidant capacity, but in Gypsy, the addition of BL or UV-B affected the antioxidant capacity and PSII photochemical efficiency more effectively than in the Pomegranate Lace, likely due to deeper penetration in leaves and lower reflectance. Thus, long-term low-intensity UV-B radiation acts as a regulatory spectral cue that differentially modulates photosynthetic and antioxidant pathways. Its integration with blue light enables cultivar-specific optimization of photochemical resistance and metabolic resilience.

## 1. Introduction

Light functions as both the primary energy source for photosynthesis and a regulatory factor controlling plant growth, development and stress physiology. These regulatory effects are mediated through photoreceptors, including phytochromes, cryptochromes, phototropins and the UV-B receptor UVR8, which initiate signaling processes via the central regulatory components COP1 and HY5 [1,2,3,4,5]. Through the combined action of these photoreceptors, plants adjust their photosynthetic performance, antioxidant capacity and the synthesis of protective metabolites in response to the spectral composition of incident light.

Cryptochromes activated by blue light promote chlorophyll accumulation, regulate the expression of genes associated with photosynthesis and contribute to antioxidant protection through suppression of COP1 activity and stabilization of HY5 [6,7]. UVR8 mediates perception of UV-B radiation and induces the biosynthesis of flavonoids and other phenolic compounds by activating genes such as *CHS*, *PAL* and *FLS*. Moderate UV-B intensities enhance the formation of these metabolites, whereas excessive UV-B exposure leads to degradation of the D1 protein and inhibition of photosystem II [8,9,10,11,12].

The responses of plants to UV-B radiation depend not only on UV-B intensity but also on the surrounding spectral environment. The concept of spectral balance describes the dependence of UV-B effects on the relative proportions of red, far-red, blue and photosynthetically active radiation, which determine the activation state of phytochromes, cryptochromes and UVR8 [13,14,15]. Consequently, many physiological and morphological responses observed under UV-B conditions may represent photomorphogenic adjustments to altered spectral ratios rather than direct UV-B-induced damage. Subsequent studies have shown that signaling through UVR8 interacts with phytochrome and cryptochrome pathways, and that the magnitude of UV-B responses is influenced by leaf optical properties, including the accumulation of phenolic compounds and anthocyanins in the epidermis [16,17,18,19]. These compounds regulate UV-B transmission to the mesophyll and thereby modulate the efficiency of UVR8 activation.

The metabolic and physiological effects of UV-B radiation are also determined by the duration and timing of exposure. Short-term or moderate UV-B irradiation applied under controlled conditions can increase the content of phenolic metabolites without negatively affecting plant growth [20]. Nocturnal UV-B exposure has been reported to modify the circadian regulation of antioxidant processes, indicating the existence of UVR8 activity in the absence of visible light. UV-B effects further depend on environmental conditions such as temperature, water availability and visible-light intensity, which influence plant stress responses and metabolic adjustments [14,15,21,22].

Despite extensive research on acute UV-B responses, considerably fewer studies have focused on continuous or long-term exposure to low-intensity UV-B, particularly when UV-B is supplied by narrowband LEDs. Such sources emit UV-B radiation without producing ozone and allow prolonged activation of UVR8 with a relatively low risk of oxidative damage [23,24]. These conditions are relevant for controlled-environment agriculture, where spectral parameters can be precisely regulated.

Red-leaf lettuce cultivars are suitable for experimental comparisons because they differ in leaf morphology, surface curvature and pigment distribution, which influence internal light gradients and the efficiency of epidermal screening [25]. Variability in anthocyanin localization and leaf structure may lead to differences in UV-B penetration and therefore in the degree of UVR8 activation. Although direct comparative data on UV-B optical properties are limited, available information indicates that leaf structural organization can affect the balance between photochemical activity and the formation of protective metabolites [26,27]. It remains insufficiently understood how continuous low-intensity UV-B interacts with white or blue background lighting in cultivars exhibiting contrasting optical traits.

The objective of the present study was to investigate the effects of continuous low-intensity UV-B applied over a 24 h period on photosynthetic activity, antioxidant responses and morphophysiological traits in two red-leaf lettuce cultivars differing in leaf structure and pigmentation. A further objective was to determine how preliminary cultivation under white or blue light influences plant responses to prolonged UV-B exposure. The working hypothesis was that leaf morphological features, including surface curvature, epidermal reflectance and the spatial arrangement of anthocyanin pigments, determine cultivar-specific UV-B sensitivity by altering internal light gradients and the efficiency of epidermal screening. Identification of spectral conditions that enhance the accumulation of phenolic compounds and maintain stable photosynthetic performance is relevant for optimizing LED-based lighting strategies in controlled-environment agriculture.

## 2. Results

### 2.1. Plant Morphology and Light Microscopy

**Figure 1:**
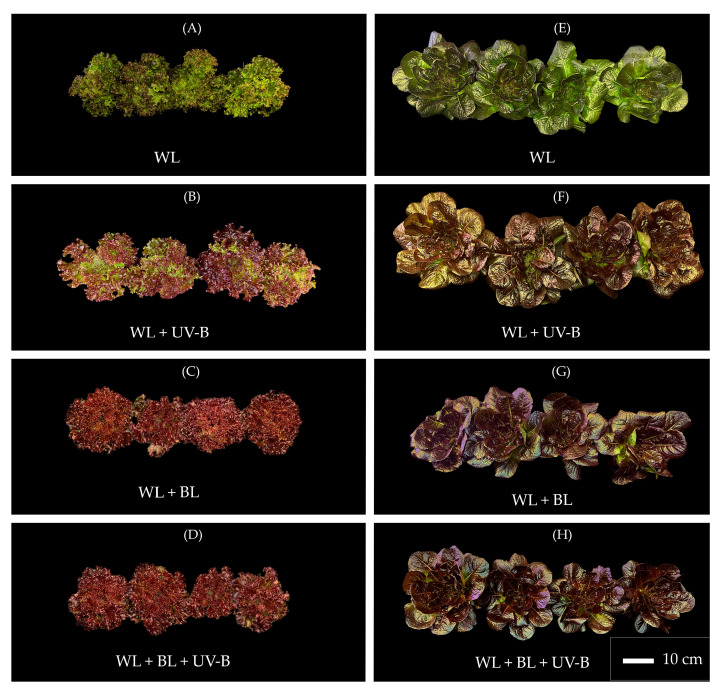
Morphological appearance of Pomegranate Lace (**A**–**D**) and Gypsy (**E**–**H**) cultivars grown under different light treatments: white light (WL), white + UV-B (WL + UV-B), white + blue light (WL + BL), and white + blue + UV-B (WL + BL + UV-B). The plants were photographed after 48 h of exposure. The scale bar represents 10 cm.

The Gypsy cultivar presented a smooth leaf surface, uniform pigmentation, and a compact rosette morphology. Under all the spectral treatments, the plants maintained structural integrity and a uniform leaf arrangement, indicating stable morphogenesis and sustained photosynthetic functionality (Figure 1E–H). Supplementation with blue and UV-B radiation increased the total phenolic content by approximately two- to threefold compared with that of WL alone in both cultivars, whereas no visible photodamage or leaf deformation was observed (Figure 1E,H; Table 1).

**Figure 2:**
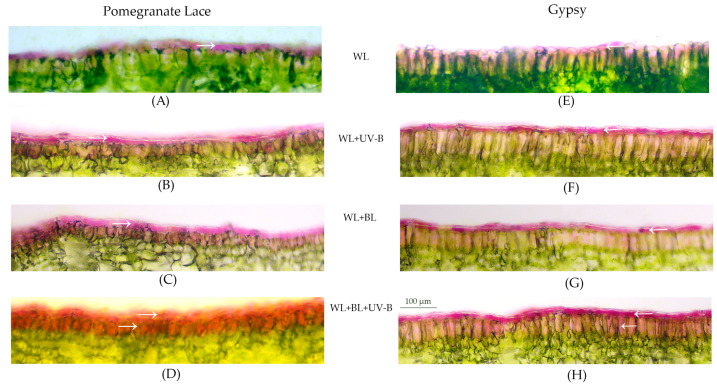
Transverse sections of leaf blades from two *Lactuca sativa* L. cultivars—Pomegranate Lace (**A**–**D**) and Gypsy (**E**–**H**)—after 48 h of exposure to different spectral treatments: white light (WL), white light supplemented with UV-B (WL + UV-B), white light combined with blue light (WL + BL), and their combination (WL + BL + UV-B). The anthocyanin-containing layer is indicated by white arrows. Light microscopy, vital sections. Scale bar = 100 µm.

In contrast, the Pomegranate Lace cultivar presented a distinctly folded leaf surface and heterogeneous anthocyanin distribution and was predominantly localized on the adaxial epidermis (Figure 2A–E, Table 1). Continuous UV-B exposure, particularly in combination with blue light (WL + BL + UV-B), resulted in a pronounced increase in anthocyanin accumulation and increased leaf curvature (Figure 1D and Figure 2D, Table 1).

In Pomegranate Lace, the adaxial epidermis presented a pronounced anthocyanin layer that varied in thickness and intensity depending on the light treatment. Under WL, the epidermal anthocyanin band was thin and pale, forming a discontinuous layer above the palisade parenchyma (Figure 2A). Exposure to UV-B (WL + UV-B) markedly intensified pigmentation, producing a continuous, dark-red layer approximately 28 µm thick (Figure 2B, Table 1). Under WL + BL, the anthocyanin zone moderately thickened and became more homogeneous, whereas the combined treatment of WL + BL + UV-B resulted in the greatest accumulation of pigments, resulting in the formation of a dense, uniform layer up to ~43 µm in size (Figure 2D).

In *Gypsy*, the epidermal anthocyanin layer was thinner and less intensely pigmented across all the treatments. Under WL, a faint pink color (~20 µm) was observed, which slightly intensified under UV-B exposure (WL + UV-B) (Figure 2E, Table 1). The addition of blue light (WL + BL) produced a moderate increase in pigment thickness and uniformity, reaching ~30 µm (Figure 2G, Table 1). Under WL + BL + UV-B, the epidermal anthocyanin layer remained compact but distinctly brighter, forming a uniform band along the leaf surface (Figure 2H). Compared with Pomegranate Lace, the epidermal response in *Gypsy* was less pronounced, indicating efficient but controlled pigment synthesis that supports photoprotection without compromising light transmission to the mesophyll (Figure 2, Table 1).

### 2.2. Contents of Photosynthetic Pigments, Activities of Low-Molecular-Weight Antioxidants and Amounts of Phenolic Compounds

The prestart parameters reflected the physiological state of the plants before the onset of experimental illumination and served as the baseline for assessing spectral effects. The 0 h point was used as the experimental baseline for analyzing subsequent temporal changes.

In the Pomegranate Lace plants (Table 2), the initial pigment concentrations were markedly lower than those in Gypsy, with a Chl *a* of approximately 4.7 mg g^−1^ DW and a Chl *b* of approximately 1.4 mg g^−1^ DW, reflecting cultivar-specific pigmentation intensity. After 24 h under WL + BL + UV-B, the contents of Chl *a*, Chl *b*, and carotenoids increased relative to the initial and WL values (reaching values of 6.25, 1.77, and 1.64 mg g^−1^ DW, respectively), indicating increased pigment biosynthesis. By 48 h, these differences became more pronounced: the Chl *a* and Chl *b* contents under WL + BL + UV-B increased to 8.18 and 2.86 mg g^−1^ DW, respectively, exceeding those under all the other treatments, while the carotenoid content also reached its maximum (1.79 mg g^−1^ DW).

The total phenolic content in Pomegranate Lace ranged from 0.42 mg GAE g^−1^ FM (prestart) to 1.20 mg GAE g^−1^ FM under WL + BL + UV-B, indicating a nearly threefold increase. Similarly, the antioxidant capacity (TEAC) increased from 3.26 to 9.06 μmol TE g^−1^ FM during the 48 h exposure, confirming the activation of phenolic metabolism and antioxidant defenses. No visible photodamage or deformation of the lamina was observed (Figure 1).

In the Gypsy plants (Table 2), the prestart pigment levels were substantially higher than those in the Pomegranate Lace plants, with the Chl *a* concentration exceeding 8 mg g^−1^ DW and the Chl *b* concentration approaching 3 mg g^−1^ DW. At 0 h, both pigments exhibited a transient decrease, with Chl *a* dropping to slightly above 5 mg g^−1^ DW and Chl *b* to approximately 1.7 mg g^−1^ DW. After 24 h under WL, pigment accumulation increased, with Chl *a* increasing to more than 13 mg g^−1^ DW and Chl *b* exceeding 4.5 mg g^−1^ DW. High pigment levels remained under WL + BL + UV-B, with Chl *a* remaining above 11 mg g^−1^ DW, but by 48 h, it declined below the WL value (Table 2). By 48 h, the Chl *a* content under WL reached its highest value (above 14 mg g^−1^ DW), whereas under WL + UV-B, it was noticeably lower. The carotenoid content increased relative to the 0 h WL level under both WL + BL and WL + BL + UV-B at 24 h; however, by 48 h, the WL + BL + UV-B treatment showed a significant reduction compared with WL, while WL + UV-B displayed only a decreasing trend. The total phenolic content also increased under these two treatments, reaching values above 1 mg GAE g^−1^ FM. TEAC showed a more than twofold increase at 0 h, with the maximum detected under WL + BL + UV-B.

### 2.3. Gas Exchange and Photosynthetic Activity

For the Pomegranate Lace plants (Table 3), the initial photosynthetic rate was approximately 6 µmol CO_2_ m^−2^ s^−1^, with corresponding moderate values of transpiration, stomatal conductance and respiration. At the beginning of the experiment under white light, photosynthesis declined to less than half of the initial level, whereas respiration increased, indicating short-term adjustment to the new light conditions. Supplementation with blue light nearly restored photosynthesis to the prestart value and reduced respiration, reflecting more efficient energy use.

After 24 h, photosynthesis under white light remained low, whereas UV-B addition increased it to approximately twice the WL value. The highest stomatal conductance (exceeding 300 mmol m^−2^ s^−1^) and transpiration were recorded under WL + BL and WL + BL + UV-B, although respiration also increased. After 48 h, photosynthesis under WL and WL + UV-B decreased to approximately 1 µmol CO_2_ m^−2^ s^−1^, whereas WL + BL resulted in the highest photosynthetic rate. Under WL + BL + UV-B, photosynthesis decreased relative to its 24 h value but remained higher than under WL, although lower than under WL + BL. Stomatal conductance showed no significant change across treatments, indicating that gas-exchange regulation was maintained rather than suppressed.

In the Gypsy plants (Table 3), all the prestart parameters were higher than those in Pomegranate Lace: photosynthesis exceeded 7.5 µmol CO_2_ m^−2^ s^−1^, transpiration was above 2 mmol H_2_O m^−2^ s^−1^, and stomatal conductance surpassed 100 mmol m^−2^ s^−1^. At the beginning of the experiment, exposure to WL reduced photosynthesis to less than 3.5 µmol CO_2_ m^−2^ s^−1^. BL increased photosynthesis and transpiration, whereas respiration remained stable.

After 24 h, photosynthesis under WL + UV-B nearly doubled relative to that under WL alone. WL + BL resulted in the greatest transpiration and stomatal opening (above 200 mmol m^−2^ s^−1^). The combination of WL + BL + UV-B resulted in intermediate photosynthesis with moderately increased respiration. After 48 h, photosynthesis decreased under WL and WL + UV-B, whereas WL + BL maintained the highest values (approximately 2.5 µmol CO_2_ m^−2^ s^−1^). The WL + BL + UV-B regime produced moderate reductions in photosynthesis, stomatal conductance and transpiration, reflecting balanced energy use during prolonged exposure.

### 2.4. Chlorophyll Fluorescence Parameters

For the cultivar Pomegranate Lace (Table 4), the effective quantum yield of PSII (Y(II)) remained relatively stable across treatments, in the range of 0.531-−0.553. The highest Y(II) values were observed under WL + BL (0.552) and WL + BL + UV-B (0.553), whereas a slight decrease occurred under WL + UV-B (0.531). Nonphotochemical quenching (Y(NPQ)) remained low under most treatments (0.143–0.150) and increased only under WL + BL + UV-B (0.175), indicating moderate activation of regulated thermal dissipation. The quantum yield of nonregulated energy loss (Y(NO)) decreased from 0.319 under WL to 0.272 under WL + BL + UV-B, suggesting reduced uncontrolled excitation losses and improved energy use efficiency in PSII. The photochemical quenching coefficient (qL) was highest under WL (0.541) and decreased slightly under UV-B illumination alone (0.428), whereas under the combined treatments, it stabilized near 0.51, reflecting the maintenance of the redox balance of Q_A_ and stable electron transport. Overall, the data indicate that the addition of blue and UV-B light to Pomegranate Lace induced mild photoprotective adjustments without disrupting PSII function or photochemical efficiency.

For cultivar Gypsy (Table 4), Y(II) under WL was 0.460, increasing significantly under WL + UV-B (0.530) and WL + BL + UV-B (0.533) but decreasing under WL + BL (0.442). The Y(NPQ) level was minimal under WL + UV-B (0.144) and reached its maximum under WL + BL + UV-B (0.200), indicating the activation of controlled heat dissipation. The Y(NO) parameter decreased markedly from 0.425 under WL to 0.267 under WL + BL + UV-B, reflecting reduced uncontrolled losses of excitation energy. The qL coefficient increased under WL + UV-B (0.488) and WL + BL (0.514) but decreased to 0.428 under WL + BL + UV-B, suggesting the dynamic regulation of PSII reaction center openness to balance excitation and electron transport. Taken together, these changes indicate that in Gypsy, the combination of blue and UV-B light promotes efficient photochemical energy utilization and photoprotection under continuous illumination.

For both cultivars, the maximum quantum efficiency (F_v_/F_m_) remained stable throughout the experiment, fluctuating between 0.80 and 0.85, confirming the absence of photoinhibitory stress and indicating that the applied spectral treatments did not impair the maximal PSII efficiency. 

### 2.5. Morphometric Characteristics and Biomass

The morphometric and biomass parameters of the Pomegranate Lace plants were generally lower than those of the Gypsy plants, reflecting varietal differences in photomorphogenic plasticity and light adaptation. Under white light (WL), the Pomegranate Lace plants had a mean fresh mass of approximately 69 g, including approximately 51 g in leaves and 18 g in roots, with a total leaf area close to 775 cm^2^ (Table 5).

The addition of UV-B radiation (WL + UV-B) caused no significant changes, although a slight increase in root mass (approximately 21 g) was recorded, suggesting a weak stimulatory effect of short-wavelength light on root growth.

Exposure to white and blue light (WL + BL) significantly reduced plant and leaf biomass (to approximately 50 g and 33 g, respectively) and decreased the total leaf area to approximately 388 cm^2^.

The combination of blue and UV-B light (WL + BL + UV-B) resulted in the most pronounced decline in growth parameters, with plant and leaf masses of approximately 46 g and 25 g, respectively, and the leaf area decreased to approximately 231 cm^2^. The dry mass also decreased to approximately 1.8 g, and the plant height decreased from approximately 11 cm under WL to 8–9 cm under the combined treatment, indicating that growth inhibition was associated with increased photoprotective investment (Table 5).

In the Gypsy plants, the morphometric and biomass parameters were consistently greater across all the treatments, confirming their higher growth potential and greater tolerance to spectral variation. Under WL, the total plant mass reached approximately 125 g, including approximately 99 g in leaves and 25 g in roots, with a total leaf area around 2080 cm^2^, a height of about 16 cm, and a rosette diameter near 28 cm (Table 5).

The addition of UV-B radiation (WL + UV-B) produced a slight, nonsignificant increase in total and leaf mass (approximately 135 g and 107 g, respectively), suggesting a weak growth-promoting effect of low-intensity UV-B.

Under WL + BL, both the plant and leaf biomass decreased (to approximately 82 g and 64 g, respectively), accompanied by a reduction in the leaf area to approximately 1390 cm^2^, the plant height to around 13 cm, and the rosette diameter to approximately 25 cm (Table 5).

The combined WL + BL + UV-B treatment induced similar trends, with moderate reductions in plant and leaf mass (about 85 g and 63 g, respectively) and a further decrease in leaf area to approximately 1240 cm^2^. The dry mass decreased from about 7.8 g under WL to approximately 4.7 g under WL + BL + UV-B. The root biomass remained stable at about 22 g, indicating the maintenance of belowground allocation patterns under continuous low-level UV-B exposure (Table 5).

## 3. Discussion

The results revealed that long-term low-intensity UV-B exposure in combination with visible light induced different restructurings of photosynthetic, photochemical, and biochemical processes in the *L. sativa* L. cultivars Gypsy and Pomegranate Lace, which differed in terms of leaf morphology and pigmentation patterns. Unlike most studies where UV-B was applied only during daytime hours [8,11], in this study, exposure lasted 48 h, including the night period, which made it possible to evaluate the effect of constant activation of the UVR8 receptor in the absence of photochemical activity and identify the possible role of night signals in maintaining the antioxidant potential and photostability of plants.

Although the applied photosynthetic photon flux density (PPFD) of 290 μmol m^−2^ s^−1^ represents moderate illumination, it falls within the optimal growth zone for *L. sativa*. Studies have demonstrated that lettuce reaches photosynthetic light saturation at approximately 200–250 μmol m^−2^ s^−1^ and that further increases in irradiance do not significantly increase CO_2_ assimilation or biomass accumulation [28,29]. Therefore, the light regime used in this study provided nonlimiting conditions for photosynthesis and did not impose low-light stress.

Moreover, maintaining irradiance only slightly above the saturation point creates favorable conditions for detecting regulatory UV-B responses. Under moderate PAR, UVR8-dependent signaling remains highly responsive because blue/UV-A photoreceptors (cryptochromes, phototropins) do not reach maximal activation [9]. This facilitates the manifestation of UV-induced metabolic and photochemical adjustments, allowing clear separation between photoprotective regulation and high-light acclimation. Thus, the selected light environment combines optimal photosynthetic performance with increased sensitivity to UV-B-mediated signaling, enabling precise assessment of cultivar-specific adaptive strategies. Importantly, the magnitude and character of the UV-B responses observed here are consistent with the classical “spectral balance” concept established by Caldwell and colleagues, which emphasizes that UV-B responses must be interpreted within the broader context of interacting visible-light photoreceptors and biologically weighted spectral composition [13,14,15,22]. According to this framework, the relative proportions of PAR, blue light and UV-B affect the activation hierarchy of phytochromes, cryptochromes and UVR8, thereby regulating the photomorphogenic versus stress-related components of UV responses.

Exposure to WL + BL increased the content of photosynthetic pigments and increased the photosynthetic activity of both cultivars. In *Gypsy*, this stimulation was sustained and accompanied by greater carotenoid accumulation, whereas in Pomegranate Lace, it was transient due to uneven light distribution caused by leaf morphology (Table 1). *Gypsy* saw increased stomatal conductance and the photosynthetic rate while maintaining a stable respiration rate, indicating the efficient use of absorbed energy and the absence of signs of stress overload. *Pomegranate Lace* presented a less pronounced and short-lived increase in the photosynthetic rate, which is consistent with its leaf morphology—a folded surface and spatially heterogeneous anthocyanin pigmentation that produces localized gradients of light intensity and increases multiple scattering within the mesophyll [30]. Thus, blue light activated cryptochrome-dependent pathways, increasing photochemical activity, but the degree of response was determined by the leaf anatomical structure.

UV-B radiation alone produced contrasting effects. In Gypsy, photosynthetic pigments remained at control levels, but the rate of photosynthesis decreased moderately (Table 2), whereas nonenzymatic antioxidant activity (ABTS) and total phenolic compounds increased, indicating that photosynthetic downregulation was compensated for by increased antioxidant metabolism, maintaining PSII stability. These changes, along with stable Y(II) and q_L_ values with moderate increases in Y(NPQ) (Table 4), indicate the activation of photoadaptation mechanisms, including an enhanced xanthophyll cycle and phenolic metabolism. Thus, UV-B acts as a regulatory rather than damaging factor, maintaining photochemical stability while increasing antioxidant capacity.

In the Pomegranate Lace cultivar, UV-B exposure was accompanied by an increase in Y(II) and q_L_, with a moderate increase in Y(NPQ), reflecting a redistribution of energy between photochemical utilization and controlled thermal dissipation. An increase in the content of phenolic compounds and antioxidant activity (Table 2) confirms the activation of the UVR8-dependent pathway and the transcription factor HY5, which regulates the biosynthesis of flavonoids and other antioxidants [9]. Importantly, however, the conclusions regarding UVR8–HY5 involvement are based on well-established mechanistic models from previous molecular studies rather than on direct gene expression measurements in the present work and are therefore interpreted here as supported hypotheses consistent with physiological patterns. Thus, in both cultivars, low-intensity UV-B radiation acted primarily as a signaling factor; however, Gypsy demonstrated adaptive stabilization of photosynthesis, whereas Pomegranate Lace demonstrated a compensatory antioxidant response with partial limitations in photochemical efficiency. This compensatory type in Pomegranate Lace is linked to enhanced epidermal pigmentation and reduced photon penetration.

The most pronounced effect was observed with a combination of blue and UV-B light. Under these conditions, Gypsy presented significant increases in chlorophyll a and b, carotenoid, and phenolic compound concentrations; increased photosynthetic activity; and high Y(II) and q_L_ levels, with a moderate increase in Y(NPQ) (Table 3). This finding indicates a coordinated interaction between cryptochrome- and UVR8-dependent signaling cascades. This interaction increased both pigment biosynthesis and antioxidant defense, maintaining photochemical efficiency over 48 h (Table 2, Table 3 and Table 4). Such interactions have been extensively documented in previous studies, where blue-light receptors increase pigment biosynthesis, whereas UV-B stimulates phenylpropanoid metabolism, collectively supporting antioxidant defense and photochemical stability [31,32]. This interaction ensures a balance between light absorption, photochemical energy utilization, and redox homeostasis, preventing photodestructive processes. It is likely that constant nocturnal UVR8 activation maintains basal HY5 activity and antioxidant metabolite synthesis [33], which contributes to maintaining photostability and energy balance under continuous light conditions.

In Pomegranate Lace, the same combination of spectra also caused an accumulation of phenolic compounds and an increase in antioxidant activity but was accompanied by a decrease in photosynthesis and an increase in respiration (Table 2 and Table 3). A decrease in Y(II) and an increase in Y(NPQ) (Table 4) reflect a shift in the energy balance toward thermal dissipation. This finding indicates that excess energy was diverted to nonphotochemical quenching as a protective response, which is consistent with the observed pigment thickening and reduced leaf area (Table 1, Table 2, Table 3 and Table 4). This response is typical for cultivars with high anthocyanin pigmentation, where tissue shielding reduces the efficiency of photon utilization, limiting photochemical productivity while maintaining antioxidant potential [34,35].

The morphometric parameters (Table 5) confirmed these differences. In *Gypsy,* plant weight, leaf area, and dry mass percentage were maintained at control levels, indicating stable growth and efficient energy utilization (Table 5). In Pomegranate Lace, a decrease in leaf area and dry mass was observed, whereas tissue moisture remained unchanged, suggesting a redistribution of metabolic resources toward defense processes (Table 5). Therefore, photosensory responses are determined not only by the light spectrum but also by leaf morphology, which influences light distribution in the mesophyll and, consequently, the balance between photochemistry and defense responses.

A comparison with the literature data revealed that long-term low-intensity UV-B radiation acts as an adaptive stimulus, increasing antioxidant activity without inhibiting photosynthesis. In daytime experiments with relatively high UV-B doses, photodegradation and a decrease in biomass were observed in lettuce and cruciferous plants [8,11], whereas 24 h irradiation combined with blue light, as in the present study, maintained PSII stability and supported plant growth.

Thus, it can be assumed that daytime UV-B exposure promotes the regulation of photochemical processes, whereas nighttime UV-B exposure maintains the activity of UVR8-HY5-dependent pathways responsible for the synthesis of phenolic and antioxidant metabolites. This interpretation is consistent with ecological and photobiological analyses showing that UV-B can perform both daytime and nocturnal regulatory functions depending on the spectral context [14,15]. This ensures a functional separation of the roles of light: during the day, energy is directed toward photosynthesis, whereas at night, it is directed toward maintaining antioxidant protection and restoring the structural components of photosystems.

The data obtained demonstrate that Gypsy exhibits an integrative adaptive response characterized by the maintenance of photosynthetic activity and redox homeostasis, whereas *Pomegranate Lace* exhibits a predominantly protective–compensatory response in which energy is redistributed toward antioxidant processes. Consequently, long-term low-intensity UV-B radiation combined with blue light can be considered a controlled spectral lighting system capable of enhancing the resilience and phytochemical quality of foliar crops without reducing their photosynthetic productivity.

## 4. Materials and Methods

### 4.1. Experimental Design and Plant Material

**Figure 3:**
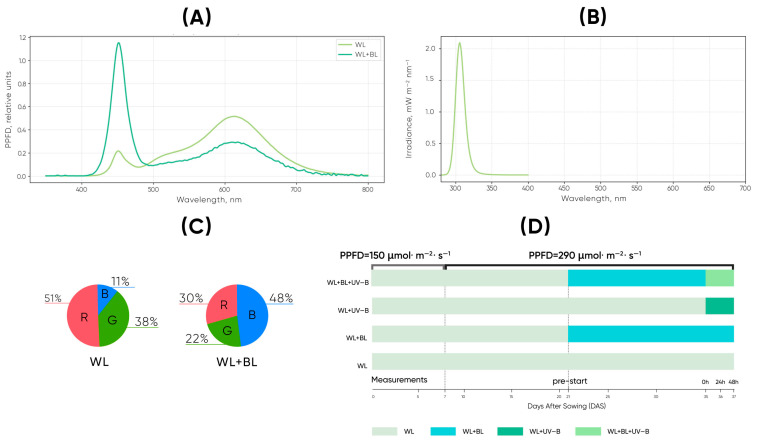
(**A**) Emission spectra of the white light (WL) and white + blue light (WL + BL) treatments measured as relative photon flux density (RFPD, µmol m^−2^ s^−1^ nm^−1^). (**B**) UV-B emission spectrum showing a narrow and well-defined peak within the 280–320 nm range, characteristic of the applied UV-B lamps. (**C**) Relative contributions of red (R), green (G), and blue (B) light components in the WL and WL + BL spectra, demonstrating the increased blue fraction under WL + BL conditions. (**D**) Experimental layout and light treatment scheme. The plants were first grown under white light (WL; PPFD 150 µmol m^−2^ s^−1^) for the initial 7 days after sowing (DAS). After this period, the PPFD increased to 290 µmol m^−2^ s^−1^. Blue light (BL) supplementation was introduced at 21 DAS, and UV-B supplementation was added at 35 DAS, resulting in four distinct light treatments: WL, WL + BL, WL + UV-B, and WL + BL + UV-B. Sampling was conducted immediately before the initiation of each treatment (pre-start) and then at 0, 24, and 48 h after exposure began.

*Lactuca sativa* L. Pomegranate Lace (https://gossortrf.ru/registry/gosudarstvennyy-reestr-selektsionnykh-dostizheniy-dopushchennykh-k-ispolzovaniyu-tom-1-sorta-rasteni/granatovye-kruzheva-salat/ (accessed on 1 November 2025)) and Gypsy (https://gossortrf.ru/registry/gosudarstvennyy-reestr-selektsionnykh-dostizheniy-dopushchennykh-k-ispolzovaniyu-tom-1-sorta-rasteni/dzhipsi-salat/ (accessed on 1 November 2025)) were used as the study objects. These cultivars differ in terms of leaf morphology, anthocyanin pigmentation patterns, and growth characteristics. Seeds from a single batch were germinated in a climate-controlled chamber until the cotyledon stage and then transferred to Hoagland nutrient solution. All the plants were cultivated under controlled environmental conditions: an air temperature of 22 ± 1 °C, a relative humidity of 60–65%, and a CO_2_ concentration of 400 ppm (Figure 3).

During the first 7 days after sowing (DAS), the seedlings were grown under white LED illumination with a photosynthetic photon flux density (PPFD) of 150 µmol m^−2^ s^−1^. The white light was provided by LEDFARM 40.0× LED modules (Ledceter, Belarus). From day 8 to day 35, the total PPFD increased to 290 µmol m^−2^ s^−1^. At this stage, two spectral conditions were applied: (1) white light only (WL) and (2) white light supplemented with blue light (WL + BL). Additional blue light (peak ≈ 450 nm) was supplied by 30 W Feron LL-903 (41522) LED lamps (Feron, Russia), with the white component proportionally reduced to maintain a constant PPFD of ~290 µmol m^−2^ s^−1^. The plants were allowed a 7-day adaptation period to these spectral regimes before UV-B exposure began.

On day 35, UV-B irradiation was introduced, resulting in four treatments: WL, WL + BL, WL + UV-B, and WL + BL + UV-B. For the UV-B treatments, the plants were exposed to continuous 24 h UV-B radiation at 30 ± 3 mW m^−2^ for 48 h. UV-B was provided by a ULTRAVAX B-PRO UV-B LED lamp (Gorshkoff, Russia). The lamp emitted a narrow-band spectrum with a peak at 309–312 nm and a spectral range of approximately 295–325 nm. Because UV-B responses depend on wavelength-specific biological effectiveness rather than irradiance alone, the UV-B spectrum was characterized using an Ocean Optics USB2000+ spectroradiometer. The spatial uniformity of illumination across the plant surfaces was maintained within ±5%, and the accompanying UV-A fraction did not exceed 10%. For the combination treatments (WL + BL + UV-B), the WL–BL spectrum was measured under a 16 h photoperiod, while UV-B was supplied continuously.

To contextualize the applied UV-B dose, long-term natural UV-B measurements in Eastern Europe indicate seasonal averages of ~19.8 kJ m^−2^ day^−1^ in summer, decreasing to ~9.9 and ~2.2 kJ m^−2^ day^−1^ in spring and autumn, respectively [36] (Figure 3).

In the present study, continuous irradiance of 0.03 W m^−2^ produced a daily UV-B dose of 2.59 kJ m^−2^ day^−1^, corresponding to 0.078 µmol m^−2^ s^−1^ or 6780 µmol m^−2^ day^−1^. Although comparable in absolute energy to autumn levels, the biological impact is substantially lower because the UV-B LED lacks shorter wavelengths (<305 nm), which have disproportionately higher biological weighting factors according to classical UV-B action spectra [22,37]. Thus, UV-B treatment represents a low-intensity, signaling-level stimulus appropriate for assessing sustained UVR8-mediated photobiological responses without inducing acute photodamage.

Sampling for physiological and biochemical analyses was performed at four time points relative to UV-B initiation: pre-start, 0 h, 24 h, and 48 h. Plant age was monitored throughout the experiment.

Physiological and biochemical analyses were conducted on days 21, 34, 36, and 38 of growth, and the time from seed germination was recorded. For each lighting treatment, at least ten biological replicates were used per variety.

### 4.2. Chlorophyll Fluorescence Measurement

Chlorophyll fluorescence measurements were performed using an IMAGING-PAM fluorometer (Walz, Germany). Prior to the measurements, the plants were dark-adapted for 30 min to ensure the complete relaxation of photosynthetic electron transport. After this adaptation period, a saturating light pulse was applied to determine the maximum fluorescence yield. The leaves were then kept in darkness for 1 min, followed by exposure to actinic light for 5 min with saturating light pulses applied at defined intervals to record fluorescence parameters. Three leaves from the second tier were used for each treatment. The leaves were positioned in front of the camera at a distance of 7 cm, and six circular regions of interest (radius 0.86 mm, area 2.35 mm^2^) were selected per leaf. Depending on the treatment, the total number of recorded regions of interest per variant varied from 50 to 100, providing average values representative of the entire leaf surface. The measuring, actinic, and saturating lights were produced by blue LEDs (450 nm). The actinic light intensity was 220 µmol photons m^−2^ s^−1^, the measuring light intensity was 0.5 µmol photons m^−2^ s^−1^, and the saturating pulse intensity and duration were 5000 µmol photons m^−2^ s^−1^ and 800 ms, respectively. Data acquisition and analysis were performed using ImagingWin v.2.41a software (Walz, Germany). The fluorescence parameters were calculated according to [38]. The maximal PSII quantum yield (F_v_/F_m_) was calculated as (F_m_ − F_0_)/F_m_ in dark-adapted leaves, where Fm and F_0_ represent the maximal and minimal fluorescence, respectively. The effective PSII quantum yield (Y(II)) was calculated as (Fm′ − Fs)/Fm′, where Fm′ is the maximal fluorescence and Fs is the steady-state fluorescence of light-adapted leaves. The coefficient of photochemical quenching (q_L_), which estimates the proportion of open PSII reaction centers, was determined as [(Fm′ − Fs)/(Fm′ − F_0_′)] × (F_0_′/F_s_). Nonphotochemical quenching (NPQ), which reflects regulated energy dissipation as heat, was calculated as F_m_/F_m_′ − 1. The quantum yield of nonregulated energy dissipation (Y(NO)), representing passive energy loss, was calculated as 1/[NPQ + 1 + q_L_ × (F_m_/F_0_ – 1)], and the quantum yield of regulated energy dissipation (Y(NPQ)), indicating photoprotective heat dissipation, was calculated as 1 − Y(II) − Y(NO).

### 4.3. Measurement of Photosynthetic Parameters

Gas exchange parameters were measured on fully expanded leaves using a Ciras-3 portable infrared gas analyser (PP Systems, USA). Measurements were performed at a controlled photosynthetic photon flux density of 290 μmol m^−2^ s^−1^, a leaf temperature of 25 °C, a relative humidity of 60%, and a reference CO_2_ concentration of 400 ppm, which corresponds to ambient atmospheric conditions. The system’s flow rate and boundary-layer conductance were maintained according to the manufacturer’s calibration settings to ensure stable chamber conditions. The net photosynthetic rate (Pn), stomatal conductance (gs), transpiration (E), and dark respiration rate (Resp) were determined after the leaf reached a steady state within the chamber.

### 4.4. Photosynthetic Pigments

The contents of chlorophyll *a,* chlorophyll *b*, and carotenoids were determined spectrophotometrically via the methods of [39]. The samples (0.2 g fresh weight) were extracted in 80% acetone; the optical density was measured at wavelengths of 663, 645, and 470 nm. Pigment concentrations were calculated via the methods of Lichtenthaler (1987) [39] and expressed as mg g^−1^ DW.

### 4.5. Determination of Phenolic Compounds

The total phenolic content was determined via the Folin–Ciocalteu method [40] using gallic acid as a standard. The optical density was measured at 725 nm, and the results are expressed as mg gallic acid per gram of fresh weight (mg GAE g^−1^).

### 4.6. Assessment of Antioxidant Capacity

Antioxidant activity was assessed by the ability of the extracts to scavenge the ABTS•^+^ radical [41]. The radical cation was generated by the reaction of ABTS (7 mM) with potassium persulfate (2.45 mM) for 16 h at 25 °C. The working solution was adjusted to an optical density of 0.70 ± 0.02 at 734 nm. A total of 0.03 mL of the plant extract was added to 3 mL of the ABTS•^+^ solution, the mixture was incubated for 6 min, and the decrease in absorbance was measured. The activity was expressed as Trolox equivalents (μmol TE g^−1^ fresh weight).

### 4.7. Light Microscopy

Leaf samples were collected from the middle portion of fully expanded leaves after 48 h of exposure to different spectral treatments. Small fragments (approximately 5 × 5 mm) were excised from the interveinal area and immediately transferred to distilled water. All the samples were processed within 2–3 min after excision to minimize dehydration and pigment redistribution. Preparations were examined under vital conditions to preserve tissue integrity, vacuolar anthocyanin localization, and epidermal optical properties.

Cross sections of the leaf blade were prepared manually using a fresh disposable razor blade, with cuts made perpendicular to the lamina surface to ensure consistent section thickness. The tissue fragments were mounted in water on glass slides and covered with 18 × 18 mm cover slips.

Microscopic observations were performed using a Leica DM2000 LED light microscope (Leica Microsystems, Germany) equipped with a Leica ICC50 HD digital camera. All imaging was carried out at room temperature (22–23 °C) under constant ambient humidity (45–55%). The illumination intensity and color temperature of the LED source were kept constant across the treatments, and identical magnification settings (10× and 40× objectives) were used for all the samples. Images were captured with LAS EZ software (v3.4.0) using fixed exposure time, gain, and white-balance settings to enable direct comparison of tissue pigmentation and morphology. Anatomical features, including the thickness of the adaxial epidermis and the anthocyanin-containing layer, were qualitatively assessed, and representative micrographs were selected for figure preparation.

### 4.8. Statistical Data Processing

All the data were statistically processed via Python (v3.10) with the libraries *pandas*, *numpy*, *scipy*, *statsmodels*, and *pingouin*. The biochemical, gas exchange, and morphometric parameters are presented as the means ± standard deviations (SD). Normality and homogeneity of variance were verified via the Shapiro–Wilk and Levene tests, respectively. Since the data met the assumptions of a normal distribution and equal variance, one-way ANOVA was applied within each cultivar and time point, followed by Tukey’s HSD test (α = 0.05) to determine significant differences among lighting treatments. Statistically homogeneous groups were visualized via small letters according to Piepho’s insert–absorb algorithm. For imaging the PAM chlorophyll fluorescence data, which did not conform to a normal distribution, the Kruskal–Wallis test was used, followed by Dunn’s post hoc test with Holm correction (α = 0.05). The results are expressed as the means ± SD. The sample sizes were as follows: biochemical traits—*n* = 3; gas exchange—*n* = 6; morphological traits—*n* = 10 (except leaf area at 48 h, *n* = 3); and imaging PAM fluorescence—*n* = 56–100, depending on cultivar and treatment. The anthocyanin layer thickness was quantified directly in ToupView (v4.11) using a calibrated scale. For each treatment and cultivar, six cross-section images were analyzed, with 15 measurement points per image (90 measurements per treatment). The data met the assumptions of normality and equal variance; therefore, one-way ANOVA followed by Tukey’s HSD test (α = 0.05) was applied. Values are presented as means ± SD.

## 5. Conclusions

Long-term low-intensity UV-B radiation (30 mW m^−2^) induces spectral-dependent restructuring of photosynthetic and antioxidant processes in *Lactuca sativa* L. cultivars with different leaf morphologies and pigmentation. This effect is likely influenced by the interaction between cryptochrome-dependent and UVR8-associated signaling mechanisms, as well as by leaf morphophysiological traits that modulate light distribution and the photochemical load.

In the Gypsy cultivar, which has uniform pigmentation and a smooth leaf surface, the combination of blue and UV-B signals enhanced photosynthetic stability and increased quantum yield and light-use efficiency while maintaining a balanced distribution between photochemical and nonphotochemical energy pathways. The increased accumulation of phenolic compounds and increased antioxidant activity suggest coordinated adjustments between photochemistry and redox regulation, supporting the formation of a stable photoadaptive state under continuous light.

In the Pomegranate Lace cultivar, which is characterized by pronounced anthocyanin accumulation and a folded leaf morphology, continuous low-intensity UV-B exposure induced a redistribution of absorbed energy toward nonphotochemical dissipation and an increase in respiratory activity. The observed increase in nonphotochemical quenching together with a moderate increase in phenolic metabolites indicates a compensatory adjustment, in which redox homeostasis is maintained by limiting photochemical efficiency and reinforcing antioxidant protection.

The results demonstrated that 24 h low-intensity UV-B predominantly acts as a regulatory stimulus, influencing the balance between photosynthetic activity and antioxidant metabolism. The sustained production of antioxidant metabolites under continuous irradiation suggests that UV-B perception during the nocturnal period contributes to maintaining protective metabolic activity when photochemical processes are inactive.

Overall, the efficiency of acclimation to prolonged UV-B appears to depend on leaf structural traits and on the capacity of the plant to coordinate photosensory inputs at the whole-organism level. The Gypsy cultivar presented a more integrated adaptive response, characterized by the maintenance of photosynthetic performance and PSII stability together with an increased antioxidant capacity. These findings indicate that the morphological and optical properties of leaves play a decisive role in determining cultivar-specific strategies for UV-B acclimation and may be useful for selecting lettuce genotypes capable of combining stable productivity with enhanced stress tolerance under controlled lighting conditions.

## Figures and Tables

**Table 1:** Thickness of the epidermal anthocyanin layer, µm.

Cultivar	Light	Anthocyanin Layer Thickness
Pomegranate Lace	WL	21.6 ± 2.5 c
	WL + UV-B	27.7 ± 2.9 b
	WL + BL	41.8 ± 4.6 a
	WL + BL + UV-B	42.9 ± 4.7 a
Gypsy	WL	20.8 ± 3.8 b
	WL + UV-B	28.7 ± 3.9 a
	WL + BL	22.8 ± 2.5 b
	WL + BL + UV-B	29.8 ± 4.2 a

The values represent means ± SD *(n* = 90). Different lowercase letters indicate statistically significant differences between light treatments within the same cultivar at α = 0.05. Statistical comparisons were performed for the 48 h time point using one-way ANOVA followed by Tukey’s HSD test.

**Table 2:** Chlorophylls, carotenoids, total phenols and antioxidant capacity in leaves of two lettuce cultivars under different light treatments.

Cultivar	Time	Light	Chl *a*	Chl *b*	Carotenoids	Total Phenols	TEAC
Pomegranate Lace	prestart	WL	4.68 ± 0.62	1.44 ± 0.20	1.44 ± 0.19	0.42 ± 0.10	3.26 ± 0.78
	0	WL	5.08 ± 0.25 a	1.54 ± 0.04 a	1.46 ± 0.01 a	0.52 ± 0.08 b	3.69 ± 0.77 b
		WL + BL	3.74 ± 0.49 b	1.13 ± 0.14 b	1.03 ± 0.16 b	0.78 ± 0.12 a	5.81 ± 0.86 a
	24	WL	5.28 ± 0.84 ab	1.60 ± 0.26 a	1.31 ± 0.22 ab	0.68 ± 0.12 b	4.83 ± 0.75 b
		WL + UV-B	3.57 ± 0.47 b	1.04 ± 0.11 b	0.93 ± 0.17 b	0.80 ± 0.09 ab	6.17 ± 0.52 ab
		WL + BL	4.20 ± 0.30 ab	1.44 ± 0.01 ab	1.08 ± 0.14 ab	1.02 ± 0.08 a	6.50 ± 0.10 ab
		WL + BL + UV-B	6.25 ± 1.49 a	1.77 ± 0.31 a	1.64 ± 0.39 a	0.85 ± 0.13 ab	6.77 ± 1.08 a
	48	WL	5.05 ± 0.47 b	1.76 ± 0.05 b	1.22 ± 0.07 c	0.62 ± 0.07 b	4.85 ± 0.68 b
		WL + UV-B	4.06 ± 0.25 c	1.28 ± 0.10 c	1.01 ± 0.07 d	0.92 ± 0.24 ab	6.85 ± 1.64 ab
		WL + BL	5.82 ± 0.39 b	1.92 ± 0.12 b	1.45 ± 0.08 b	1.00 ± 0.07 ab	7.44 ± 0.42 ab
		WL + BL + UV-B	8.18 ± 0.25 a	2.86 ± 0.12 a	1.79 ± 0.01 a	1.20 ± 0.31 a	9.06 ± 2.64 a
Gypsy	prestart	WL	8.28 ± 1.08	2.71 ± 0.18	2.63 ± 0.28	0.47 ± 0.02	3.47 ± 0.14
	0	WL	5.14 ± 0.64 a	1.70 ± 0.15 a	1.43 ± 0.16 a	0.76 ± 0.26 a	5.17 ± 1.22 b
		WL + BL	5.47 ± 0.16 a	1.83 ± 0.03 a	1.44 ± 0.06 a	1.43 ± 0.48 a	15.62 ± 1.38 a
	24	WL	13.53 ± 1.08 a	4.74 ± 0.78 a	3.24 ± 0.01 a	1.00 ± 0.15 a	7.37 ± 1.17 a
		WL + UV-B	11.60 ± 1.30 a	3.82 ± 0.53 a	2.84 ± 0.25 a	0.69 ± 0.03 b	5.17 ± 0.52 b
		WL + BL	10.94 ± 1.40 a	3.35 ± 0.40 a	2.86 ± 0.36 a	0.64 ± 0.13 b	4.77 ± 0.73 b
		WL + BL + UV-B	11.84 ± 2.13 a	3.68 ± 0.54 a	2.95 ± 0.51 a	1.02 ± 0.15 a	7.44 ± 0.63 a
	48	WL	14.55 ± 3.06 a	4.66 ± 1.11 a	3.51 ± 0.68 a	0.60 ± 0.09 b	4.62 ± 0.62 b
		WL + UV-B	8.93 ± 2.41 ab	2.86 ± 0.62 ab	2.20 ± 0.58 ab	1.26 ± 0.19 a	9.50 ± 1.77 a
		WL + BL	12.50 ± 1.11 a	4.00 ± 0.40 a	3.04 ± 0.25 a	0.74 ± 0.06 b	5.53 ± 0.06 b
		WL + BL + UV-B	7.18 ± 1.24 b	2.62 ± 0.35 b	1.65 ± 0.32 b	1.34 ± 0.19 a	10.00 ± 1.74 a

Contents of chlorophyll *a* (Chl *a*, mg g^−1^ DW), chlorophyll *b* (Chl *b*, mg g^−1^ DW), carotenoids (mg g^−1^ DW), total phenols (mg GAE g^−1^ FW), and antioxidant capacity (TEAC, µmol Trolox g^−1^ FW) in Pomegranate Lace and Gypsy leaves exposed to different light treatments (white light—WL, white + blue light—WL + BL, white + UV-B—WL + UV-B, and white + blue + UV-B—WL + BL + UV-B) at different exposure times (prestart, 0, 24, and 48 h). The values are expressed as the means ± SD (*n* = 3). Lowercase letters indicate statistically significant differences between light treatments within the same time point and cultivar at α = 0.05. Statistical comparisons were performed separately for each time point (0, 24, 48 h) using one-way ANOVA followed by Tukey’s HSD test.

**Table 3:** Photosynthetic parameters of in leaves of two lettuce cultivars under different light treatments.

Cultivar	Time	Light	Pn	E	g_S_	Resp
Pomegranate Lace	prestart	WL	6.09 ± 1.03	1.94 ± 0.39	69.67 ± 10.75	−1.80 ± 0.20
	0	WL	2.61 ± 1.09 b	1.03 ± 0.33 a	41.50 ± 16.03 a	−3.05 ± 0.25 b
		WL + BL	5.12 ± 0.07 a	1.40 ± 0.30 a	48.00 ± 11.00 a	−1.32 ± 0.23 a
	24	WL	1.73 ± 0.08 c	1.70 ± 0.10 b	78.50 ± 3.50 b	−2.03 ± 0.21 b
		WL + UV-B	3.46 ± 0.17 a	1.67 ± 0.21 b	90.33 ± 10.21 b	−1.47 ± 0.35 b
		WL + BL	2.27 ± 0.15 b	3.43 ± 0.32 a	301.00 ± 9.00 a	−6.40 ± 0.26 a
		WL + BL + UV-B	2.35 ± 0.05 b	3.23 ± 0.23 a	290.00 ± 3.00 a	−6.05 ± 0.55 a
	48	WL	1.03 ± 0.15 c	1.50 ± 0.10 c	106.67 ± 12.22 c	−7.03 ± 0.15 a
		WL + UV-B	1.07 ± 0.15 c	1.80 ± 0.20 bc	118.67 ± 14.57 bc	−5.73 ± 0.29 b
		WL + BL	2.30 ± 0.20 a	3.47 ± 0.35 a	293.00 ± 21.52 a	−6.60 ± 0.30 ab
		WL + BL + UV-B	1.53 ± 0.13 b	2.33 ± 0.21 b	180.67 ± 27.63 b	−6.63 ± 0.47 ab
Gypsy	prestart	WL	7.65 ± 0.67	2.34 ± 0.35	101.50 ± 23.80	−2.03 ± 0.21
	0	WL	3.37 ± 0.01 b	1.07 ± 0.15 a	41.00 ± 6.08 a	−2.13 ± 0.31 a
		WL + BL	4.12 ± 0.29 a	1.75 ± 0.50 a	66.80 ± 24.38 a	−2.00 ± 0.20 a
	24	WL	2.91 ± 0.89 ab	1.18 ± 0.15 c	53.75 ± 6.99 c	−2.02 ± 0.10 b
		WL + UV-B	4.95 ± 0.79 a	1.75 ± 0.31 bc	117.00 ± 13.23 b	−1.10 ± 0.10 a
		WL + BL	2.50 ± 0.26 b	3.40 ± 0.22 a	203.33 ± 23.71 a	−1.40 ± 0.51 ab
		WL + BL + UV-B	3.03 ± 0.93 ab	2.36 ± 0.30 b	116.20 ± 20.19 b	−3.15 ± 0.05 c
	48	WL	1.65 ± 0.15 b	1.35 ± 0.05 c	75.50 ± 2.50 d	−2.05 ± 0.05 a
		WL + UV-B	1.27 ± 0.32 b	2.27 ± 0.25 b	152.00 ± 5.29 b	−8.50 ± 0.50 c
		WL + BL	2.50 ± 0.30 a	3.30 ± 0.20 a	203.67 ± 19.50 a	−5.53 ± 0.45 b
		WL + BL + UV-B	2.33 ± 0.15 a	1.60 ± 0.20 bc	114.00 ± 7.81 c	−4.77 ± 0.15 b

Plants were exposed to different light treatments: white light (WL), white + blue light (WL+BL), white + UV-B (WL+UV-B), and white + blue + UV-B (WL + BL + UVB) at various exposure times (prestart, 0, 24, and 48 h). Net photosynthetic rate (Pn, μmol CO_2_ m^−2^ s^−1^); transpiration rate (E, mmol H_2_O m^−2^ s^−1^); stomatal conductance (gs, mmol H_2_O m^−2^ s^−1^); respiration rate (Resp, μmol CO_2_ m^−2^ s^−1^). The data are presented as the means ± SD (*n* = 6). Lowercase letters indicate post hoc comparisons within each sampling time. The prestart time point is shown for reference and is not included in the statistical testing.

**Table 4:** Chlorophyll fluorescence parameters in leaves of two lettuce cultivars under different light treatments.

Cultivar	Parameter	WL	WL + UV-B	WL + BL	WL + BL + UV-B
Pomegranate Lace	Y(II)	0.549 ± 0.018 a	0.531 ± 0.017 a	0.552 ± 0.018 a	0.553 ± 0.011 a
	Y(NPQ)	0.143 ± 0.024 b	0.150 ± 0.012 b	0.144 ± 0.021 b	0.175 ± 0.022 a
	Y(NO)	0.319 ± 0.058 ab	0.319 ± 0.014 a	0.304 ± 0.030 ab	0.272 ± 0.025 b
	q_L_	0.541 ± 0.078 a	0.428 ± 0.030 b	0.509 ± 0.044 ab	0.517 ± 0.047 ab
Gypsy	Y(II)	0.460 ± 0.074 b	0.530 ± 0.032 a	0.442 ± 0.032 b	0.533 ± 0.010 a
	Y(NPQ)	0.148 ± 0.039 ab	0.144 ± 0.016 b	0.173 ± 0.033 ab	0.200 ± 0.012 a
	Y(NO)	0.425 ± 0.136 a	0.326 ± 0.024 b	0.412 ± 0.103 a	0.267 ± 0.008 c
	q_L_	0.361 ± 0.084 b	0.488 ± 0.045 a	0.514 ± 0.179 a	0.428 ± 0.018 c

Plants were exposed for 48 h to different light treatments: white light (WL), white + blue light (WL + BL), white + UV-B (WL + UV-B), and white + blue + UV-B (WL + BL + UV-B). Effective quantum yield of PSII photochemistry (Y(II)); quantum yield of regulated nonphotochemical energy dissipation (Y(NPQ)); quantum yield of nonregulated energy dissipation (Y(NO)); coefficient of photochemical quenching, qL reflects the fraction of open PSII reaction centers according to the “lake” model, relative units. The data are presented as the means ± SD (*n* = 56–100). Different lowercase letters indicate statistically significant differences among treatments according to the Kruskal–Wallis test followed by the Dunn–Holm post hoc test (*p* < 0.05).

**Table 5:** Morphological parameters of two lettuce cultivars under different light treatments.

Cultivar	Parameter	WL	WL + UV-B	WL + BL	WL+BL + UV-B
Pomegranate Lace	Plant mass, g	69.40 ± 12.83 a	68.11 ± 14.73 a	50.38 ± 11.86 b	46.00 ± 6.07 b
	Fresh leaf mass, g	51.40 ± 12.47 a	47.44 ± 13.65 ab	32.50 ± 9.38 ab	24.62 ± 4.27 b
	Fresh root mass, g	18.00 ± 2.05 a	20.67 ± 3.43 a	17.88 ± 3.44 a	21.38 ± 5.53 a
	Leaf area/plant, cm^2^	775.10 ± 47.90 a	735.38 ± 7.96 a	388.38 ± 32.22 b	231.31 ± 8.44 c
	Leaf number, pcs	12.40 ± 4.72 a	13.33 ± 6.44 a	11.50 ± 1.93 a	9.88 ± 3.56 a
	Height, cm	11.25 ± 2.04 a	11.39 ± 1.27 a	8.12 ± 1.33 a	9.25 ± 0.89 a
	Diameter, cm	21.40 ± 2.37 a	21.44 ± 1.94 a	17.75 ± 3.28 a	19.50 ± 2.14 a
	Dry mass, g	3.56 ± 0.86 a	3.31 ± 0.95 ab	2.30 ± 0.66 ab	1.75 ± 0.30 b
Gypsy	Plant mass, g	124.60 ± 31.81 ab	134.70 ± 19.79 a	82.27 ± 16.23 b	84.88 ± 11.32 b
	Fresh leaf mass, g	99.20 ± 26.58 ab	106.60 ± 17.82 a	63.55 ± 13.19 ab	63.25 ± 9.21 b
	Fresh root mass, g	25.40 ± 6.87 ab	28.10 ± 3.63 a	18.73 ± 4.34 b	21.62 ± 3.02 ab
	Leaf area/plant, cm^2^	2084.07 ± 231.13 a	1818.87 ± 258.47 ab	1394.31 ± 164.66 b	1239.78 ± 237.99 b
	Leaf number, pcs	30.20 ± 6.48 a	35.20 ± 4.08 a	29.09 ± 5.43 a	29.50 ± 2.62 a
	Height, cm	16.30 ± 1.67 a	15.10 ± 0.77 a	12.98 ± 1.36 a	13.38 ± 1.85 a
	Diameter, cm	27.50 ± 2.72 a	28.60 ± 2.07 a	24.73 ± 2.20 a	23.75 ± 2.05 a
	Dry mass, g	7.85 ± 2.10 a	8.39 ± 1.40 a	4.95 ± 1.03 ab	4.71 ± 0.69 b

Plants were exposed to white light (WL), white + UV-B (WL + UV-B), white + blue light (WL + BL), and white + blue + UV-B (WL + BL + UVB). The parameters included total plant mass, fresh leaf and root mass, leaf area per plant, leaf number, plant height, stem diameter, and dry leaf mass. The morphological traits of the lettuce were measured at the end of the experiment (48 h) under different light treatments. The data are presented as the means ± SD. For leaf area, the number of biological replicates was *n* = 3; for all other traits, it was *n* = 10. Different small letters indicate significant differences between light treatments within the same cultivar according to one-way ANOVA followed by Tukey’s HSD test (α = 0.05).

## Data Availability

The datasets generated and analyzed during the current study are available from the corresponding author upon reasonable request. The data are not publicly available due to ongoing analyses and their use in related ongoing research projects.
